# Hepatic Levels of DHA-Containing Phospholipids Instruct SREBP1-Mediated Synthesis and Systemic Delivery of Polyunsaturated Fatty Acids

**DOI:** 10.1016/j.isci.2020.101495

**Published:** 2020-08-22

**Authors:** Daisuke Hishikawa, Keisuke Yanagida, Katsuyuki Nagata, Ayumi Kanatani, Yoshiko Iizuka, Fumie Hamano, Megumi Yasuda, Tadashi Okamura, Hideo Shindou, Takao Shimizu

**Affiliations:** 1 Department of Lipid Signaling, National Center for Global Health and Medicine, Shinjuku-ku, Tokyo 162-8655, Japan; 2 Life Science Core Faculty, Graduate School of Medicine, The University of Tokyo, Bunkyo-ku, Tokyo 113-0033, Japan; 3 Laboratory Animal Medicine, National Center for Global Health and Medicine, Shinjuku-ku, Tokyo 162-8655, Japan; 4 Section of Animal Models, Department of Infectious Diseases, National Center for Global Health and Medicine, Shinjuku-ku, Tokyo 162-8655, Japan; 5 Department of Lipid Science, Graduate School of Medicine, The University of Tokyo, Bunkyo-ku, Tokyo 113-0033, Japan; 6 Department of Lipidomics, Graduate School of Medicine, the University of Tokyo, Bunkyo-ku, Tokyo 113-0033, Japan

**Keywords:** Cellular Physiology, Molecular Physiology, Molecular Biology

## Abstract

Polyunsaturated fatty acids (PUFAs), such as docosahexaenoic acid (DHA) and arachidonic acid (ARA), play fundamental roles in mammalian physiology. Although PUFA imbalance causes various disorders, mechanisms of the regulation of their systemic levels are poorly understood. Here, we report that hepatic DHA-containing phospholipids (DHA-PLs) determine the systemic levels of PUFAs through the SREBP1-mediated transcriptional program. We demonstrated that liver-specific deletion of *Agpat3* leads to a decrease of DHA-PLs and a compensatory increase of ARA-PLs not only in the liver but also in other tissues including the brain. Together with recent findings that plasma lysophosphatidylcholine (lysoPC) is the major source of brain DHA, our results indicate that hepatic AGPAT3 contributes to brain DHA accumulation by supplying DHA-PLs as precursors of DHA-lysoPC. Furthermore, dietary fish oil-mediated suppression of hepatic PUFA biosynthetic program was blunted in liver-specific *Agpat3* deletion. Our findings highlight the central role of hepatic DHA-PLs as the molecular rheostat for systemic homeostasis of PUFAs.

## Highlights

• DHA-PLs' deficiency leads to liver-specific induction of PUFA biosynthetic genes
• AGPAT3-produced DHA-PLs in the liver are the partial source of DHA in the brain
• SREBP1 upregulates PUFA biosynthetic genes in response to hepatic DHA-PLs' deficiency
• Dietary DHA suppresses hepatic PUFA biosynthetic genes in an AGPAT3-dependent manner

## Graphical Abstract

**Figure:**
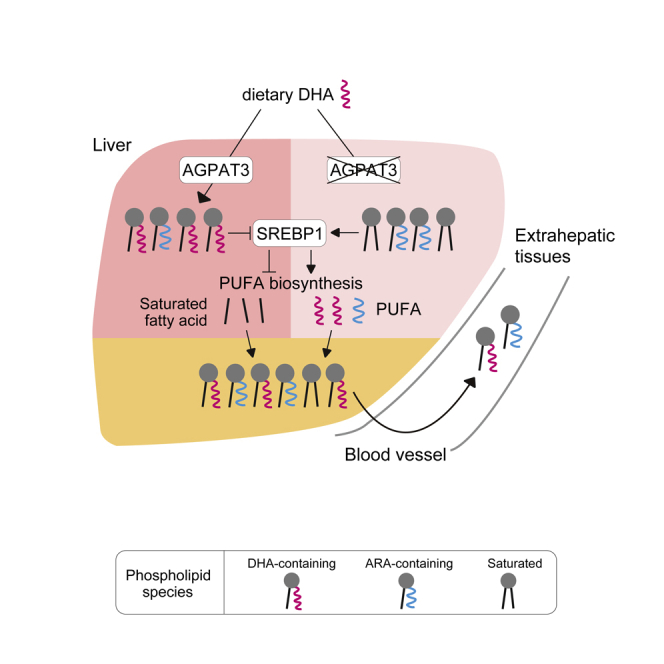

## Introduction

Recent studies have shown that besides their quantity, the qualities of fatty acids (e.g., saturated fatty acid toxicity and omega-3/omega-6 fatty acid balance) are involved in various human diseases, including metabolic syndrome, inflammatory diseases, and neuronal diseases (Bazinet and Laye, 2014; Estadella et al., 2013). Especially, omega-3 polyunsaturated fatty acids (PUFAs), such as docosahexaenoic acid (DHA), and omega-6 PUFAs, including arachidonic acid (ARA), are implicated in diverse cellular processes and in the progression of these diseases as bioactive lipid mediators, or as components of membrane phospholipids (PLs) (Harayama and Shimizu, 2020; Jump et al., 2013; Milligan et al., 2017; Shimizu, 2009; Wang and Tontonoz, 2019). PUFA-containing PLs are supposed to affect the membrane-based cellular processes, such as endo/exocytosis, and localization and functions of a number of membrane proteins by providing fluidity to the cellular membrane (Antonny et al., 2015; Harayama and Riezman, 2018). Indeed, recent studies with knockout (KO) mouse models of 1-acyl-*sn*-glycerol-3-phosphate acyltransferase 3 (AGPAT3, also known as lysophosphatidic acid acyltransferase 3, LPAAT3) and lysophosphatidylcholine acyltransferase 3 (LPCAT3) have revealed the critical roles of DHA- and ARA-containing PLs in the maintenance of photoreceptor disks, spermatogenesis, and lipoprotein secretion *in vivo* (Hashidate-Yoshida et al., 2015; Iizuka-Hishikawa et al., 2017; Rong et al., 2015; Shindou et al., 2017).

As mammals cannot synthesize DHA and ARA *de novo* because of lack of enzymes that introduce the double bond at omega-3 and 6 position, dietary intake of these PUFAs or their precursors (α-linolenic acid for DHA and linoleic acid for ARA) is required for maintenance of their systemic levels. Although DHA and ARA can be directly supplied through diet or supplements, prior studies indicate that their production from precursor fatty acids is essential to maintain the levels (Moon et al., 2009; Pauter et al., 2014). For the syntheses from precursor fatty acids, both DHA and ARA require a number of common enzymes, namely, fatty acid desaturase 1 (FADS1), FADS2, and elongation of very long fatty acid protein-5 (ELOVL5) (Jalil et al., 2019). In the case of PUFAs with >22 carbon chains, such as DHA, an additional enzyme, ELOVL2, is also required (Jalil et al., 2019). All these PUFA biosynthetic enzymes are highly expressed in the liver; therefore, the liver has been considered as a central organ for the systemic metabolism and distribution of PUFAs.

Sterol regulatory element-binding proteins (SREBPs) are the key transcription factors required for lipid metabolism in the liver (Horton et al., 2002; Scorletti and Byrne, 2013). SREBP1 controls the expression of genes encoding the enzymes involved in *de novo* fatty acid synthesis and PUFA production, whereas SREBP2 controls cholesterol synthesis-related genes. SREBP1 and SREBP2 are translated as transmembrane proteins, and proteolytic cleavage is required for their activation as transcription factors (Shimano and Sato, 2017). SREBP2-mediated regulation of cellular cholesterol levels has been well documented. When the cellular cholesterol level goes down, SREBP2 translocates from the endoplasmic reticulum to the Golgi apparatus, where it is proteolytically cleaved and activated as a major transcription factor for the biosynthesis of cholesterol (Goldstein et al., 2006). Similarly, several studies have shown that supplementation of PUFAs, but not of saturated fatty acids, negatively regulates SREBP1 (Hannah et al., 2001; Kato et al., 2008; Yahagi et al., 1999). Although these observations suggest the negative feedback regulation of PUFA levels, an overview of the precise molecular mechanisms controlling the cellular and systemic PUFA levels are still enigmatic.

Herein, we investigated the role of hepatic membrane DHA-PLs by specifically manipulating the DHA levels in the PLs without affecting the other forms of DHA, including triglycerides and cholesterol ester, using a liver-specific deletion of AGPAT3, a critical enzyme for DHA-PLs' production, in mice. We propose a model whereby systemic PUFA levels are maintained through SREBP1-mediated biosynthesis in response to excess or deficiency of hepatic DHA-PLs.

## Results

### Transcriptional Upregulation of PUFA Biosynthetic Pathway in DHA-PL-Deficient Liver

**Figure 1:**
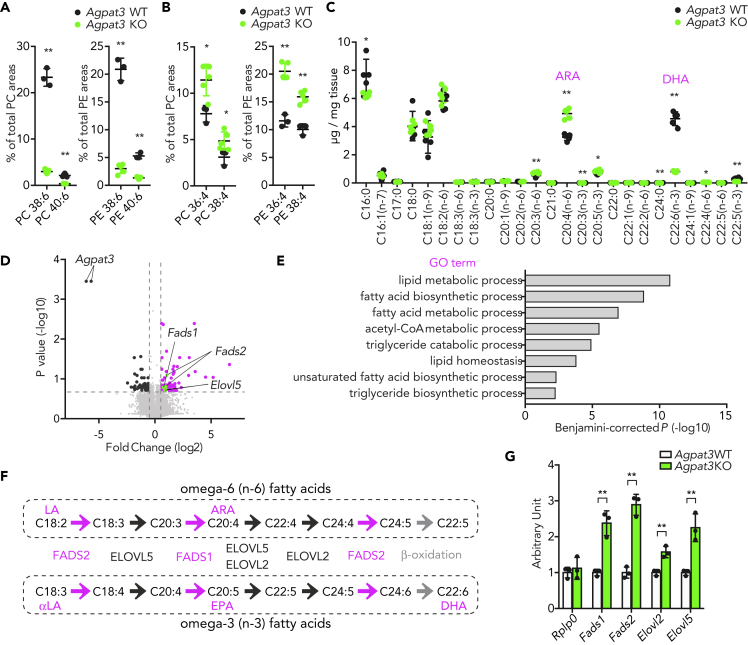
Lipidomic and Transcriptomic Changes in the Liver of *Agpat3* KO Mice (A–G) Liver samples were prepared from 10-week-old Agpat3 WT and KO mice. (A and B) Percentages of PC and PE with cumulative six (A) and four (B) double bonds in the liver (n = 4 for each group). (C) Fatty acid composition in the liver (n = 5 for each group). (D and E) DNA microarray analysis of the liver of Agpat3 WT and KO mice. (D) Volcano plot summarizes the differences in the gene expression profile in the liver of Agpat3 WT and KO mice. Differentially expressed genes (fold change >1.5, p < 0.2) are highlighted in magenta for an increase and in black for a decrease in Agpat3 KO mice. Genes involved in the synthesis of PUFA are highlighted in green (n = 4 for each group). (E) Functional annotation of upregulated genes (shown in magenta and green in Figure 1D) in the liver of Agpat3 KO mice. The top gene ontology (GO) terms from “biological processes” are shown (Benjamini-corrected p < 0.01). (F) Biosynthetic pathway for PUFAs in mammals. Fatty acid desaturation processes and enzymes are shown in magenta. Fatty acid elongation processes and enzymes are shown in black. Peroxisomal β-oxidation process is shown in gray. LA, linoleic acid; αLA, α-linolenic acid; EPA, eicosapentaenoic acid; ARA, arachidonic acid; DHA, docosahexaenoic acid. (G) Relative expression of genes involved in the synthesis of PUFAs in the liver of 1-week-old mice (n = 3 for each group). Rplp0 is used as an internal control. (A, B, C, and G) Data are shown as means ± SD. Significance is based on unpaired t test (∗p < 0.05, ∗∗p < 0.01). See also Figure S1.

We previously reported that AGPAT3 is critical for the production of DHA-PLs in the retina and testes (Iizuka-Hishikawa et al., 2017; Shindou et al., 2017). To determine whether AGPAT3 is essential for the production of DHA-PLs in the liver, we first assessed the fatty acid composition of phosphatidylcholine (PC) and phosphatidylethanolamine (PE), the most abundant species in the membrane PLs, in *Agpat3* wild-type (WT) and KO liver by liquid chromatography-tandem mass spectrometry (LC-MS/MS). Consistent with retina and testes, PCs and PEs having a total of six double bonds (mainly composed of DHA-PLs) were dramatically decreased in the liver of *Agpat3* KO mice (Figures 1A, S1A, and S1B). In contrast to the decrease of DHA-PLs, there was an increase in the levels of unsaturated PC and PE species (Figures S1A and S1B), especially those with four cumulative double bonds (mainly composed of ARA-PLs) in *Agpat3* KO liver (Figure 1B). The increase in the levels of unsaturated fatty acids in *Agpat3* KO liver was further validated by quantitative analysis of the amount of fatty acids chemically deacylated from total lipids using gas chromatography-flame ionization detection. Consistent with LC-MS/MS analyses, various PUFAs, especially ARA, were significantly increased in the liver of *Agpat3* KO mice (Figure 1C). These results led us to hypothesize that liver increases the ARA under DHA deficiency to maintain the hepatic PUFA levels. Thus, to explore the molecular mechanisms underlying the compensatory increase of ARA in *Agpat3* KO liver, we next analyzed the hepatic transcriptional profiles by DNA microarray. Gene ontology analyses indicated that the genes related to lipid metabolism, including those involved in PUFA biosynthesis, were upregulated in *Agpat3* KO liver (Figures 1D and 1E). The biosynthetic pathways for DHA and ARA share a number of enzymes, namely, FADS1, FADS2, and ELOVL5 (ELOVL2 is also required for DHA) (Figure 1F). As the long-term defect of DHA-PLs may affect the expression of these genes, we also analyzed their mRNA expression in the liver of 1-week-old WT and *Agpat3* KO mice. Consistent with adult mice (Figure S1C), mRNA levels of PUFA biosynthetic enzymes were higher in the liver of 1-week-old *Agpat3* KO mice (Figure 1G). Therefore, it is plausible that the liver possesses the feedback machinery to maintain the hepatic PUFA levels under DHA-deficient conditions by transcriptional boosting of PUFA biosynthesis.

### Liver-Specific Upregulation of Transcriptional PUFA Biosynthetic Pathway in Response to DHA-PL Deficiency

**Figure 2:**
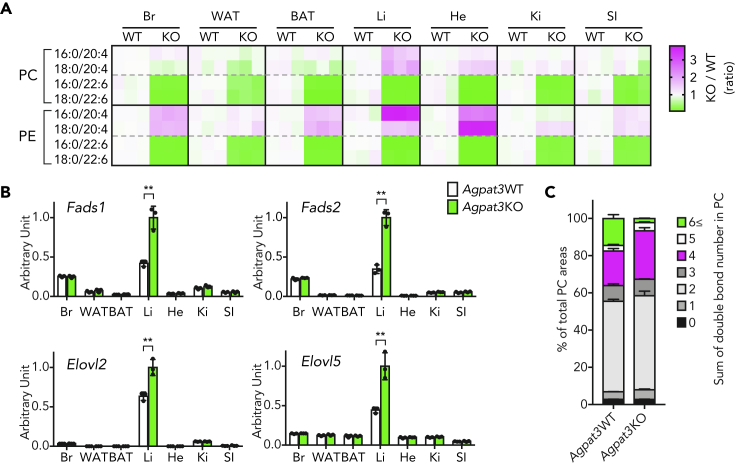
Liver-Specific Induction of the Transcription of Genes Encoding Enzymes Involved in the Biosynthesis of PUFA under Systemic DHA-PL Deficiency (A–C) Samples were prepared from 1-week-old Agpat3 WT and KO mice. (A) Heatmap shows the rational differences in each PL species in various tissues (% of total area in WT/% of total area in KO, n = 3 for each group). Br, brain; WAT, white adipose tissue; BAT, brown adipose tissue; Li, liver; He, heart; Ki, kidney; SI, small intestine. (B) Relative mRNA expression of genes involved in the biosynthesis of PUFA in various tissues (n = 3 for each group). The expression data of the liver are identical to Figure 1G. Significance is based on two-way ANOVA followed by Bonferroni's post-hoc test (∗∗p < 0.01). (C) Percentages of PC species in the plasma with different number of cumulative double bonds (n = 3 for each group). (B and C) Data are shown as means ± SD. See also Figure S2.

To investigate whether the induction of PUFA biosynthetic enzymes under DHA-deficient condition is specific to the liver, we examined both the composition of PLs and gene expression in various tissues of *Agpat3* KO mice. Although mRNA expression level of *Agpat3* differed among tissues both in adult and 1-week-old WT mice (Figure S2A and S2B), DHA-PLs were drastically decreased in all tested tissues (Figures 2A, S2C, and S2D). As in the case of the liver, the levels of ARA-PCs and ARA-PEs were increased in various tissues (Figure 2A). Contrary to the commonly observed increased ARA-PL levels in various tissues, PUFA biosynthetic enzymes were transcriptionally upregulated exclusively in the liver of *Agpat3* KO mice (Figure 2B). The liver is the central organ that supplies the fatty acids, mainly through the secretion of very low-density lipoprotein (VLDL); the elevation of ARA-containing lipids in the liver of *Agpat3* KO mice may secondarily affect the levels of ARA-PLs in various extrahepatic tissues. Indeed, the percentage of plasma PCs with four double bonds, mainly composed of ARA-PCs, was increased in the *Agpat3* KO mice (Figure 2C). Collectively, these results suggest that the liver increases the expression of PUFA biosynthetic enzymes in response to the depletion of membrane DHA-PLs and facilitates the maintenance of systemic PUFA levels through the secretion of ARA-enriched lipoproteins.

### Effect of Liver-Specific Deletion of *Agpat3* on Systemic Homeostasis of PUFAs

**Figure 3:**
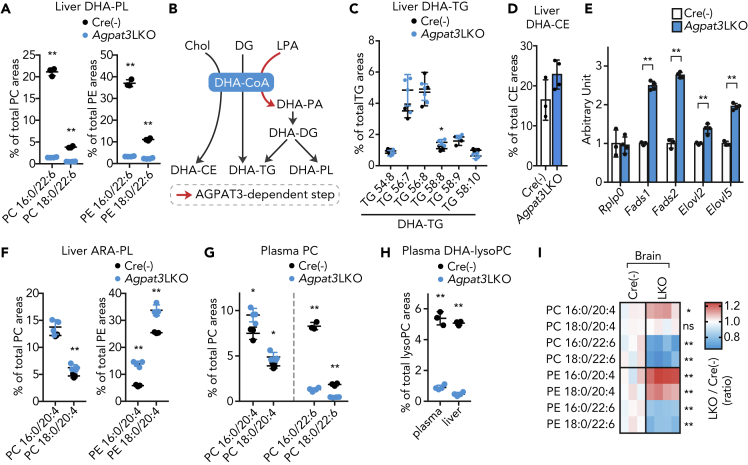
Effects of Liver-Specific Agpat3 Deficiency on Lipid Composition and Gene Expression (A–I) Samples were prepared from 2-week-old Agpat3 liver-specific KO (LKO) and Cre(−) (control) mice. (A) Percentages of DHA-PC and DHE-PE containing areas in the liver. (B) Synthetic pathways for DHA-containing lipids. Chol, cholesterol; DG, diacylglycerol; LPA, lysophosphatidic acid; PA, phosphatidic acid. (C and D) Percentages of DHA-containing TGs (C) and CE (D) areas in the liver. (E) Relative mRNA expression of genes encoding the PUFA biosynthetic enzymes involved in the liver. (F) Percentages of ARA-PC and ARA-PE areas in the liver. (G) Percentages of DHA-PCs/PE and ARA-PCs/PE areas in the plasma. (H) Percentages of DHA-lysoPC areas in the plasma and liver. (I) Heatmap shows the rational differences in the levels of each PL species in the brain (% of total area in Cre(−)/% of total area in LKO; n = 3 for Cre(−) and n = 4 for Agpat3 LKO). Each column represents data for a mouse. (A and C–H) Data are shown as means ± SD (n = 3 for Cre(−) and n = 4 for Agpat3 LKO). Significance is based on unpaired t test (∗p < 0.05, ∗∗p < 0.01, ns; no significance). See also Figure S3.

To further assess the intrinsic role of liver DHA-PL production, we generated tamoxifen-inducible liver-specific *Agpat3* KO (LKO) mice by crossing *Agpat3* floxed mice with serum albumin Cre-ERT2 mice (Schuler et al., 2004) (Figures S3A and S3B). Intragastric injection of tamoxifen at postnatal day 1 and 2 successfully achieved the liver-specific deletion of *Agpat3* in the liver (Figures S3B–S3E). As was observed in the global KO model, Alb-CreERT2^+/−^; *Agpat3*^*fl/fl*^ mice (referred to as *Agpat3* LKO) showed a drastic decrease in the levels of DHA-PLs compared with Alb-CreERT2^−/−^; *Agpat3*^*fl/fl*^ mice (control) (Figures 3A, S3F, and S3G), without marked effects on the total PL levels in the liver (Figure S3H). DHA-CoA is a substrate not only for DHA-PL but also for the DHA-containing triglycerides (DHA-TGs) and DHA-containing cholesterol ester (DHA-CE) (Figure 3B). Therefore, we analyzed the DHA-TGs and DHA-CE levels in the liver of *Agpat3* LKO mice using LC-MS/MS. Unlike DHA-PLs, the levels of DHA-TGs and DHA-CE in the liver of *Agpat3* LKO mice were almost comparable to those of control mice (Figures 3C and 3D). As liver-specific deletion of *Agpat3* did not alter the total amount of TGs in the liver (Figure S3I), these results indicate that the liver-specific deletion of *Agpat3* selectively decreased the levels of DHA-PLs, without affecting the other forms of DHA. We next assessed the effect of hepatic DHA-PL depletion on the expression of genes encoding PUFA biosynthetic enzymes in the liver of *Agpat3* LKO mice. In agreement with the observation in the global KO line (Figure 1), there was an upregulation of PUFA biosynthetic enzymes' mRNA (Figure 3E), as well as an increase in the levels of ARA-PLs (Figure 3F), in the liver of *Agpat3* LKO mice.

To examine the effect of liver DHA-PL production on extrahepatic tissues, we next analyzed the levels of plasma DHA-containing lipids in *Agpat3* LKO and control mice. Substantial reduction in the levels of DHA-PLs and DHA-lysoPC in the plasma of *Agpat3* LKO mice indicated the critical contribution of hepatic AGPAT3-dependent production of DHA-PLs on their plasma pools (Figures 3G, 3H, S3F, and S3G). Importantly, compensatory increase in the levels of ARA-PLs was also observed in *Agpat3* LKO plasma (Figures 3G and S3F, and S3G). The plasma DHA-TG levels were comparable to those in control mice (Figures S3J and S3K), whereas the DHA-CE level in the plasma was decreased in *Agpat3* LKO mice (Figure S3L), possibly due to the lecithin cholesterol acyltransferase-dependent formation of plasma CEs (Sakai et al., 1997). We then analyzed the fatty acid composition of PLs and TGs in extrahepatic tissues of *Agpat3* LKO and control mice at 2 weeks of age. The levels of PLs in the brain were substantially affected by the deletion of liver *Agpat3*; there was a decrease in the levels of DHA-PLs/TGs and an increase in the levels of ARA-PLs (Figures 3I, S3F, S3G, and S3M). Similar trend, although with a weaker effect than observed in global KO mice, was observed in other tissues from *Agpat3* LKO mice, including the brown adipose tissue, heart, and kidney (Figures S3F, S3G, and S3N). Taken together, our lipidomic analyses of *Agpat3* LKO tissues demonstrated that deficiency of hepatic DHA-PLs affects the systemic PUFA composition, especially in the brain.

### Induction of SREBP1-Dependent PUFA Biosynthetic Enzymes under DHA-PL Deficiency in the Liver

**Figure 4:**
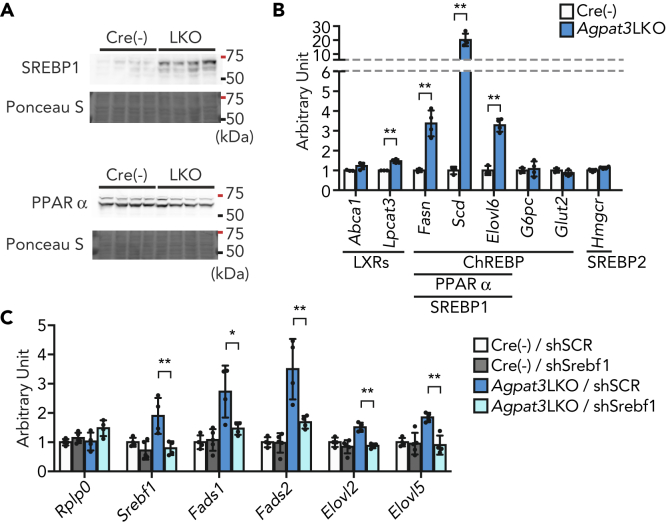
SREBP1-Mediated Upregulation of mRNAs in the Liver of Agpat3LKO Mice (A–C) Liver samples were prepared from 2-week-old Agpat3 LKO and Cre(−) mice. (A) Immunoblot analysis of the amount of SREBP1 and PPARα in the nucleus (n = 4 for each group). Ponceau S staining was as a loading control. (B) Relative mRNA expression of representative LXRs-, SREBP1-, SREBP2-, ChREBP-, and PPARα-target genes in the liver (n = 3 for Cre(−) and n = 4 for Agpat3 LKO mice). Significance is based on unpaired t test (∗p < 0.05, ∗∗p < 0.01). (C) The effect of short hairpin RNA (shRNA)-mediated Srebf1 knockdown on the expression of mRNAs encoding the PUFA biosynthetic enzymes in the liver (n = 3 for each group). Rplp0 is used as an internal control. shSCR, scrambled shRNA (control); shSrebf1, Srebf1 shRNA. Significance is based on two-way ANOVA followed by Bonferroni's post-hoc test (∗p < 0.05, ∗∗p < 0.01). (B and C) Data are shown as means ± SD. See also Figure S4.

We next tried to decipher the mechanism underlying the transcriptional induction of PUFA biosynthetic enzymes in the liver of *Agpat3* LKO mice. The transcription of the genes for these enzymes in the liver is mainly regulated by two transcription factors, SREBP1 and peroxisome proliferator-activated receptor alpha (PPARα) (Jump, 2008; Matsuzaka et al., 2002). In the liver of *Agpat3* LKO mice, the nuclear amount of SREBP1, but not of PPARα, was clearly increased (Figures 4A and S4A). In general, it is considered that SREBP1 and SREBP2 share the mechanism for nuclear localization; however, increased nuclear amount was specifically observed for SREBP1 in the liver of *Agpat3* LKO mice (Figures S4A and S4B). Consistently, the expression of SREBP1-target genes, namely, *Fasn*, *Scd*, and *Elovl6*, but not of a representative SREBP2-target gene, *Hmgcr*, was upregulated in the liver of *Agpat3* LKO mice (Figure 4B). In line with this, protein expression of insulin-induced gene 1 (INSIG1), which regulates the translocation of SREBP1 and SREBP2 (Shimano and Sato, 2017), in the liver of *Agpat3* LKO was comparable to that of control mice (Figure S4C). Liver X receptors (LXRs) and carbohydrate regulatory element-binding protein (ChREBP) can also transcriptionally control the PUFA biosynthetic enzymes (Jalil et al., 2019; Postic et al., 2007); however, their contribution to the latter is minimal in the liver of *Agpat3* LKO mice (Figure 4B).

To further validate the involvement of SREBP1 in the upregulation of PUFA biosynthetic enzymes in *Agpat3* LKO liver, we performed an *in vivo* gene knockdown experiment using an adenovirus-mediated short hairpin RNA (shRNA) expression system (Figure S4D). Three days after injection, shRNA for *Srebf1*, gene name for SERBP1, decreased the mRNA and protein expression of SREBP1 in the liver of control and *Agpat3* LKO mice (Figures 4C and S4E). The knockdown of SREBP1 blunted the induction of PUFA biosynthetic enzymes in the liver of *Agpat3* LKO mice (Figure 4C). On the other hand, we observed little or no effect of *Srebf1* knockdown on the levels of *Srebf1* and PUFA biosynthetic enzymes mRNA in control mice (Figure 4C). These results suggest that SREBP1, when compared with other transcription factors such as LXRs or PPARα, has little contribution to the basal gene expression levels of these enzymes at 2 weeks of age, whereas being critically important for compensatory mRNA upregulation during DHA-PL deficiency. Taken together, all these results indicate a major role of SREBP1 in the induction of transcription of PUFA biosynthetic enzymes under conditions of hepatic DHA-PL deficiency.

### Requirement of AGPAT3-Dependent DHA Incorporation into PLs upon Fish Oil-Mediated Suppression of Hepatic SREBP1

**Figure 5:**
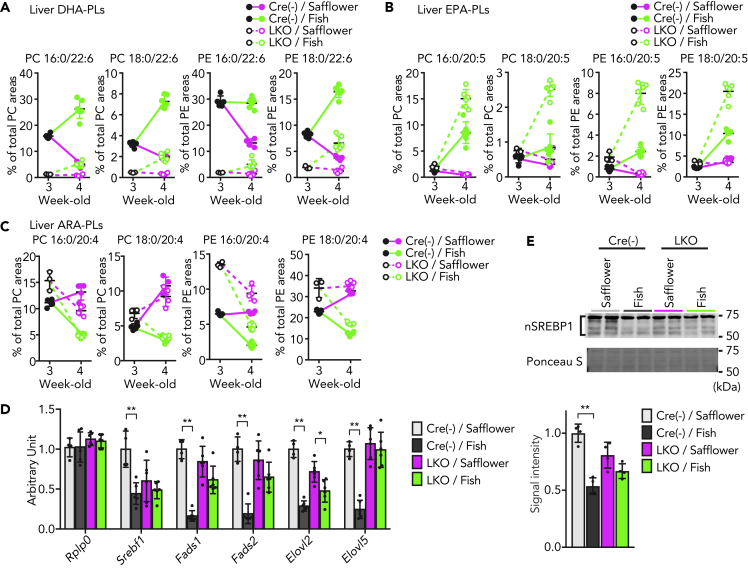
Involvement of Hepatic DHA-PLs in Fish Oil-Mediated Suppression of SREBP1 (A–C) Liver samples were prepared from 3- and 4-week-old Agpat3 LKO and Cre(−) (control) mice. (A–C) The change in the composition of DHA-PCs/PEs (A), EPA-PCs/PEs (B), and ARA-PCs/PEs (C) in the liver (n = 5 for 3-week-old Cre(−); n = 3 for 3-week-old Agpat3 LKO, n = 4 for safflower-diet-fed Cre(−) and Agpat3 LKO, and n = 6 for fish-diet-fed Cre(−) and Agpat3 LKO mice). (D and E) Liver samples were prepared from 4-week-old mice. (D) Relative mRNA expression of PUFA biosynthetic enzymes (n = 4 for safflower-diet-fed Cre(−) and Agpat3 LKO, and n = 6 for fish-diet-fed Cre(−) and Agpat3 LKO mice). (E) Upper panel; Representative image of immunoblot analysis of nuclear SREBP1 (nSREBP1) in the whole liver lysate of Agpat3 LKO and Cre(−) mice. Ponceau S staining was used as a loading control. Lower panel; Bar graph shows the signal intensity of nSREBP1, as quantified by ImageJ software. Safflower, safflower-diet; Fish, fish-diet. (A–E) Data are shown as means ± SD. (D and E) Significance is based on two-way ANOVA followed by Bonferroni's post-hoc test (∗p < 0.05, ∗∗p < 0.01). See also Figure S5 and Table S1.

Omega-3 PUFAs, especially DHA and eicosapentaenoic acid (EPA), are clinically used to reduce the levels of TGs in the liver and blood, in part by the suppression of SREBP1-mediated lipogenic transcriptional program in the liver (Bays et al., 2008; Clarke, 2001). However, the precise mechanisms of how omega-3 PUFAs inhibit the SREBP1 activity are still obscure. Therefore, we investigated whether DHA-PLs are involved in the effect of omega-3 PUFAs on hepatic SREBP1 activity *in vivo*. For this purpose, *Agpat3* LKO and control mice were fed a high-fat diet (HFD) containing omega-3 PUFA-enriched fish oil (fish diet) or calorie-matched safflower oil-containing HFD (safflower diet) for 1 week, starting at 3 weeks of age (Figure S5A and Table S1). The fish diet increased the levels of DHA- and EPA-PLs, but not ARA-PLs, in the liver of both control and *Agpat3* LKO mice (Figures 5A, 5B, and 5C). However, the levels of DHA-PLs in the *Agpat3* LKO mice were still substantially lower than in control mice (Figures 5A, 5B, S5B, and S5E). In contrast, the levels of EPA-PLs were higher in the liver of *Agpat3* LKO mice than in the control mice, which was suggestive of the specific contribution of AGPAT3 in DHA-PL production (Figure 5B). Although diet affects the fatty acid composition of TGs in the liver, the *Agpat3* genotype had no effect on the composition (Figure S5F).

We subsequently examined the hepatic mRNA expression of PUFA biosynthetic enzymes in these mice. In agreement with previous studies (Kim et al., 1999; Shang et al., 2017), the levels of SREBP1 mRNA and its downstream PUFA biosynthetic genes in the liver were clearly lower in the fish-diet-fed mice than in safflower-diet-fed groups when applied on the liver of control mice (Figure 5D). On the other hand, such reduction caused by the fish diet was less obvious in the liver of *Agpat3* LKO mice (Figure 5D). In safflower-diet-fed groups, the levels of hepatic mRNAs of PUFA biosynthetic enzymes in *Agpat3* LKO mice were comparable with the levels in control mice (Figure 5D). It may, at least in part, be due to the reduction of DHA-PL in the safflower-diet-fed control mice (Figure 5A). Consistently, the reduction of the nuclear form of SREBP1 protein by fish-diet in the liver appears to be blunted by liver-specific deletion of *Agpat3* (Figure 5E). Together with the lipidomic analyses, the suppression of the transcription of PUFA biosynthetic enzymes by omega-3 PUFA-enriched diet would require the incorporation of DHA, and not of EPA, into the membrane PLs.

## Discussion

The essential roles of DHA in various tissues have been demonstrated in genetic DHA deficiency models (Ben-Zvi et al., 2014; Harauma et al., 2017; Nguyen et al., 2014; Roqueta-Rivera et al., 2010; Stoffel et al., 2008; Stroud et al., 2009; Wong et al., 2016). We previously reported that AGPAT3 (LPAAT3) preferentially incorporates DHA into lysoPA, a common precursor of all kinds of PLs. Genetic ablation of the enzyme causes impaired spermatogenesis and blindness due to the decrease of DHA-PLs in testis and retina, respectively (Iizuka-Hishikawa et al., 2017; Shindou et al., 2017). Although the liver has been shown as one of the organs in which the membranes are most enriched in DHA (Harayama et al., 2014), the roles of DHA-PLs in the liver have not yet been clearly defined. Here, we successfully unraveled the specific role of DHA-PLs in the liver membranes, using *Agpat3* global and LKO mice. Our study demonstrates that hepatic DHA-PLs have a central role as a molecular rheostat for the regulation of systemic PUFA levels by modulating the SREBP1-mediated PUFA synthetic programs.

Among several extrahepatic organs, the most prominent effect of *Agpat3* LKO was observed on the fatty acid composition of the brain (Figures 3I and S3N). This is in agreement with recent findings in KO mice of the major facilitator superfamily domain-containing protein 2A (MFSD2A), a transporter of PUFA-containing lysoPC, showing that organs of the central nervous system, such as the brain, greatly depend on circulatory DHA-lysoPC for their DHA source (Nguyen et al., 2014). Substantial decrease in the levels of DHA-lysoPC in *Agpat3* LKO plasma indicates that the circulatory DHA-lysoPC is derived from hepatic AGPAT3. In contrast, plasma DHA-TG levels were not altered in the *Agpat3* LKO mice (Figure 3C). The relatively smaller effect of *Agpat3* on fatty acid compositions in the organs other than the brain suggests a possible contribution of DHA-TGs (in the form of lipoproteins) as sources of DHA in these organs. In addition, the different turnover rate of DHA in tissues would affect the impact of liver-specific deletion of *Agpat3* on the DHA levels in extrahepatic tissues.

A major finding of this study is that the upregulation of transcripts encoding the PUFA synthetic enzymes in *Agpat3* KO mice was seen mainly in the liver despite the consistent reduction of DHA-PLs in all other organs including the brain (Figures 2A and 2B). As these enzymes are abundantly expressed in the liver, one may think that the chromatin accessibility of sterol regulatory element in their promoter region may facilitate the liver specificity. However, this scenario cannot fully explain our observations because *Mfsd2a* KO mice, another mouse model showing brain and retinal DHA reduction, displayed the induction of SREBP1-target genes in these tissues (Chan et al., 2018; Wong et al., 2016). Currently, we do not have evidence to explain the different behaviors. As MFSD2A transports not only DHA-lysoPCs but also other fatty acid-lysoPCs, the altered levels of fatty acids other than DHA can contribute to the upregulation of SREBP1-target genes in the brain and retina of *Mfsd2a* KO mice. The detailed mechanistic link between DHA and SREBP1 activity in the brain will be revealed in the future studies by employing neuron-specific *Agpat3* KO.

SREBP1 regulates the expression of genes involved in *de novo* fatty acid synthesis concomitantly with PUFA biosynthesis (Jump et al., 2013). Along with the increased prevalence of metabolic syndrome, such as obesity and hepatic steatosis, previous studies have exclusively focused on SREBP1-mediated *de novo* lipogenesis (Moslehi and Hamidi-Zad, 2018; Shimano and Sato, 2017). Moreover, the roles of SREBP1-mediated PUFA synthesis have not been thoroughly investigated. Our observation regarding the SREBP1-mediated regulation of PUFA biosynthetic programs by responding the hepatic DHA-PL levels may indicate an autonomous regulatory mechanism for the homeostasis of PUFA levels. As the levels of unsaturated fatty acids in PLs have been shown to affect the membrane properties, such as fluidity and flexibility (Budin et al., 2018; Harayama and Riezman, 2018; Pinot et al., 2014), the increase in ARA-PLs in *Agpat3* KO mice may reflect the compensatory mechanism that allows the maintenance of membrane integrity under DHA deficiency. In this context, the liver of *Agpat3* LKO mice displayed increased levels of monounsaturated fatty acid (MUFA)-containing PLs (Figures S1 andS2) along with the upregulation of genes, such as *Fasn*, *Scd*, and *Elovl6*, which encode the enzymes for the *de novo* synthesis of MUFAs, further supporting the idea.

Although PUFAs are essential lipid components in mammals, they are sensitive to oxidation and converted to cytotoxic lipids, such as hydroperoxides or malondialdehyde (Ayala et al., 2014; Gaschler and Stockwell, 2017); their systemic levels should, therefore, be maintained properly. Previous studies have shown that supplementation of PUFAs, but not saturated fatty acids, decreases the nuclear localization of SREBP1 and its target genes (Hannah et al., 2001; Kato et al., 2008; Kim et al., 1999; Mater et al., 1999; Xu et al., 1999; Yahagi et al., 1999), suggesting the regulation of cellular PUFA levels through a feedback mechanism involving SREBP1. We demonstrated that inhibitory effects of omega-3 PUFA enriched-diet on SREBP1 are exerted mainly by DHA incorporated into PL membranes, but not by EPA (Figures 5A and 5B). The model proposed by us based on these data, whereby SREBP1 mediates the tuning of PUFA levels by responding to the DHA-PL levels in the membrane, may also contribute to preventing excessive production of PUFA. However, we do not exclude the possibility that altered fatty acid composition of PLs, other than PC and PE, in *Agpat3* KO mice affect our observation, because the direct product of AGPAT3, DHA-containing phosphatidic acid, is the common intermediate for all classes of PLs, including phosphatidylinositol and phosphatidylserine (Figure 3B).

The role of liver DHA-PLs presented here is in contrast to that of ARA-PLs, which are fundamental for the formation of hepatic lipoproteins (Hashidate-Yoshida et al., 2015; Rong et al., 2015). The reduction of ARA-PLs by genetic deletion of *Lpcat3* led to neonatal lethality because of malnutrition triggered by the failure of lipoprotein formation (Hashidate-Yoshida et al., 2015; Rong et al., 2015). As high levels of PUFA (both ARA and DHA) in PC promote TG transfer *in vitro* (Hashidate-Yoshida et al., 2015), it is proposed that decreased membrane fluidity in *Lpcat3* KO cells leads to the disrupted formation of lipoproteins and massive accumulation of intracellular TGs (Hashidate-Yoshida et al., 2015; Rong et al., 2015). However, deficiency of DHA-PLs in *Agpat3* KO mice did not exhibit these phenotypes produced by the deficiency of ARA-PLs. Although the mechanism for the difference in lipoprotein formation caused by the KO of *Agpat3* and *Lpcat3* is unknown, LPCAT3-mediated local production of ARA-PC may be specifically required for the normal lipoprotein formation, as previously proposed (Hashidate-Yoshida et al., 2015; Rong et al., 2015).

The difference in the effects of the deficiency of DHA-PLs and ARA-PLs on transcriptional programs should be noted. Unlike DHA-PL deficiency in *Agpat3* KO mice, reduction in ARA-PLs by deletion of *Lpcat3* did not induce the SREBP1-mediated transcriptional program in the liver (Rong et al., 2017). Instead, it has been demonstrated that intestine-specific *Lpcat3* KO leads to the upregulation of genes involved in cholesterol biosynthesis through the activation of SREBP2 (Wang et al., 2018). Both SREBP1 and SREBP2 are transmembrane proteins, and proteolytic cleavage-dependent release of their N terminus is required for their nuclear translocation (Horton et al., 2002). The question is how different PUFA-PLs regulate different classes of SREBPs. Although site-1 protease (S1P)- and S2P-dependent cleavage is proposed as a common model for their activation, several lines of evidences suggest distinctive mechanisms for their proteolytic activation. For instance, amino acid substitution of S1P and S2P target sequences blocks the cleavage of SREBP2, but not of SREBP1c (a major isoform of hepatic SREBP1) in mouse liver (Nakakuki et al., 2014). Therefore, S1P/S2P-independent cleavage by this unidentified protease(s) may be involved in the selective activation of SREBP1 in the liver of *Agpat3* KO mice. In such case, specific interaction of DHA-PLs, but not of ARA- or EPA-PLs, may change their conformation and activity of protease(s). Together with the selective activation of SREBP2 in *Lpcat3* KO mice, the PUFA-composition in membrane PLs may contribute to the fine-tuning of lipid metabolism through SREBPs. In addition to the protease-dependent SREBP1 activation, DHA supplementation is reported to destabilize the nuclear SREBP1 *in vitro*. Thus, SREBP1 stability may, at least in part, affect the increased nuclear SREBP1 in the liver of *Agpat3* LKO mice.

### Limitations of the Study

Although our study provides a novel and fundamental framework for the maintenance of systemic PUFA levels using global and liver-specific *Agpat3* KO mice, two issues remain to be clarified in future studies; these are liver specificity and DHA specificity for maintenance of PUFA levels. It is desirable to examine the chromatin status for SREBP1-target genes in various tissues from *Agpat3* KO mice as well as from other DHA-deficient animal models. In addition, the mechanism and the responsible proteolytic enzyme(s) have to be determined to understand how DHA deficiency in membrane PLs causes the activation of SREBP1, but not of SREBP2. As aberrant induction of SREBP1 in the metabolically abnormal situations, including tumor progression and aging, is reported (Guo et al., 2014; Ishizuka et al., 2020; Soyal et al., 2015), these studies will provide a clue to understand the basis of various diseases, including dyslipidemia, liver steatosis, atherosclerosis, inflammatory diseases, and cancers.

### Resource Availability

#### Lead Contact

Further information and requests for resources and reagents should be directed to and will be fulfilled by the Lead Contact, Takao Shimizu (tshimizu@ri.ncgm.go.jp).

#### Materials Availability

All unique reagents and animals generated in this study are available from the Lead Contact with a completed Materials Transfer Agreement.

#### Data and Code Availability

The microarray data in this manuscript have been deposited in Gene Expression Omnibus (GEO; https://www.ncbi.nlm.nih.gov/geo/). GEO accession number; GSE154724.

## Methods

All methods can be found in the accompanying Transparent Methods supplemental file.

## Supplemental Information

Document S1. Transparent Methods, Figures S1–S5, and Table S1
