# Supplementary material for: Hepatic Levels of DHA-Containing Phospholipids Instruct SREBP1-Mediated Synthesis and Systemic Delivery of Polyunsaturated Fatty Acids
Source: iScience. 2020 Aug 22;23(9):101495. doi: 10.1016/j.isci.2020.101495 (PMC7481256; doi:10.1016/j.isci.2020.101495)
Supplement: Document S1. Transparent Methods, Figures S1–S5, and Table S1 [file mmc1.pdf]

iScience, Volume *23*

Supplemental Information

Hepatic Levels of DHA-Containing Phospholipids Instruct SREBP1-Mediated Synthesis and Systemic Delivery of Polyunsaturated Fatty Acids

Daisuke Hishikawa, Keisuke Yanagida, Katsuyuki Nagata, Ayumi Kanatani, Yoshiko Iizuka, Fumie Hamano, Megumi Yasuda, Tadashi Okamura, Hideo Shindou, and Takao Shimizu

Supplemental Information

Figures S1-S5

Supplemental Figure Legends

Table S1

Transparent Methods

Supplemental References

FIGURE S1

A

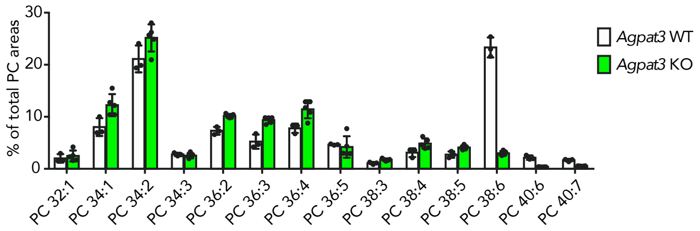

B

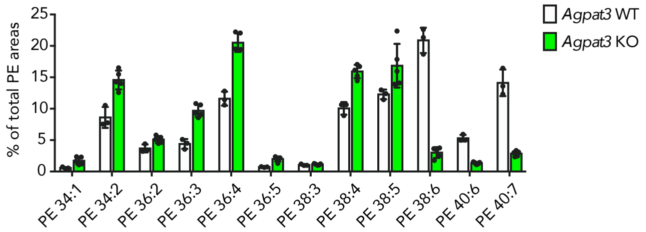

C

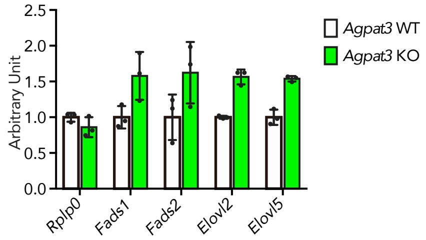

FIGURE S2

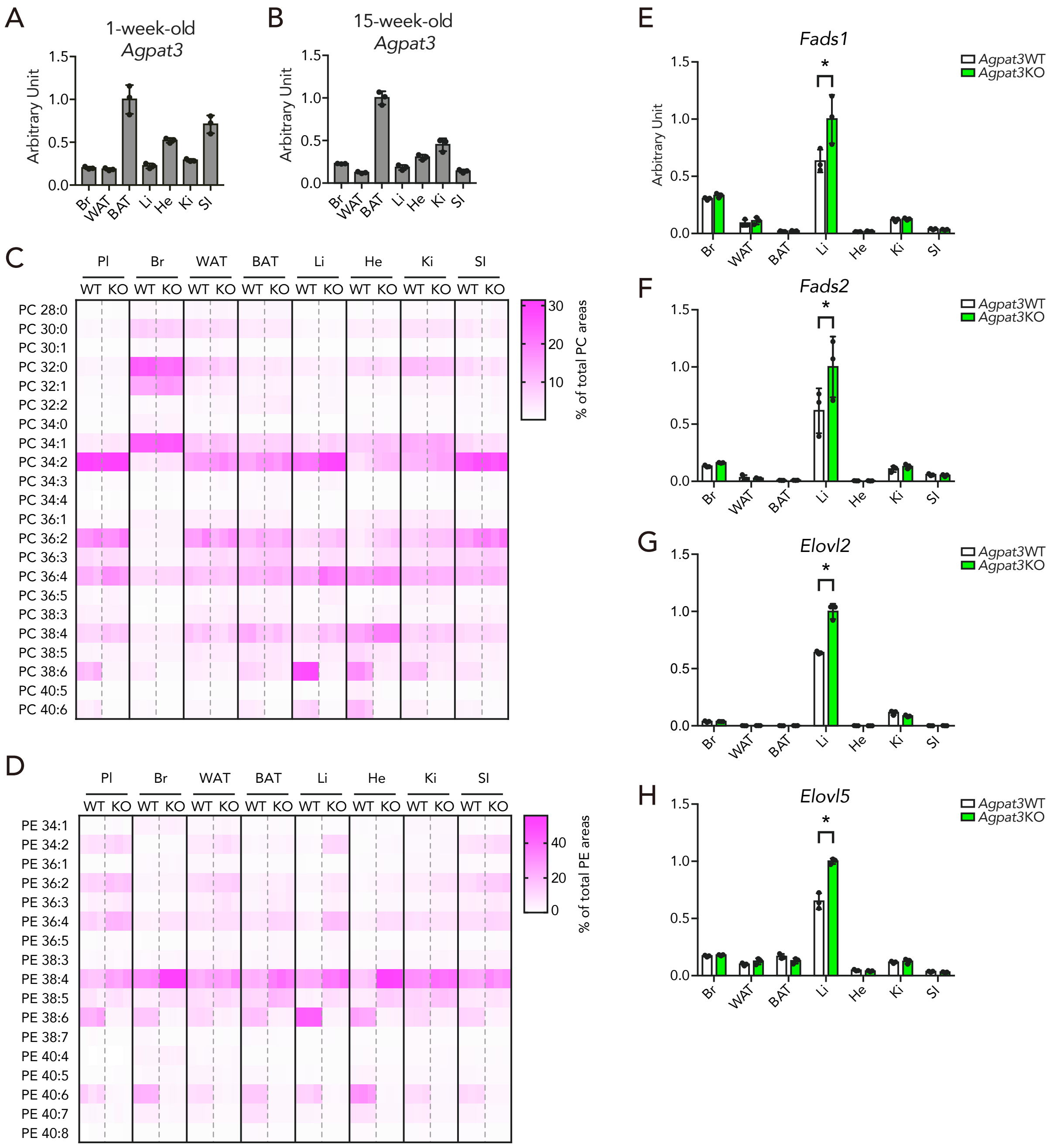
A
1-week-old
Agpat3
Arbitrary Unit
Br WAT BAT Li He Ki SI
B
15-week-old
Agpat3
C
Pl Br WAT BAT Li He Ki SI
WT KO
PC 28:0
PC 30:0
PC 30:1
PC 32:0
PC 32:1
PC 32:2
PC 34:0
PC 34:1
PC 34:2
PC 34:3
PC 34:4
PC 36:1
PC 36:2
PC 36:3
PC 36:4
PC 36:5
PC 38:3
PC 38:4
PC 38:5
PC 38:6
PC 40:5
PC 40:6
% of total PC areas
D
PE 34:1
PE 34:2
PE 36:1
PE 36:2
PE 36:3
PE 36:4
PE 36:5
PE 38:3
PE 38:4
PE 38:5
PE 38:6
PE 38:7
PE 40:4
PE 40:5
PE 40:6
PE 40:7
PE 40:8
% of total PE areas
E
Fads1
Agpat3WT
Agpat3KO
F
Fads2
G
Elovl2
H
Elovl5

FIGURE S3

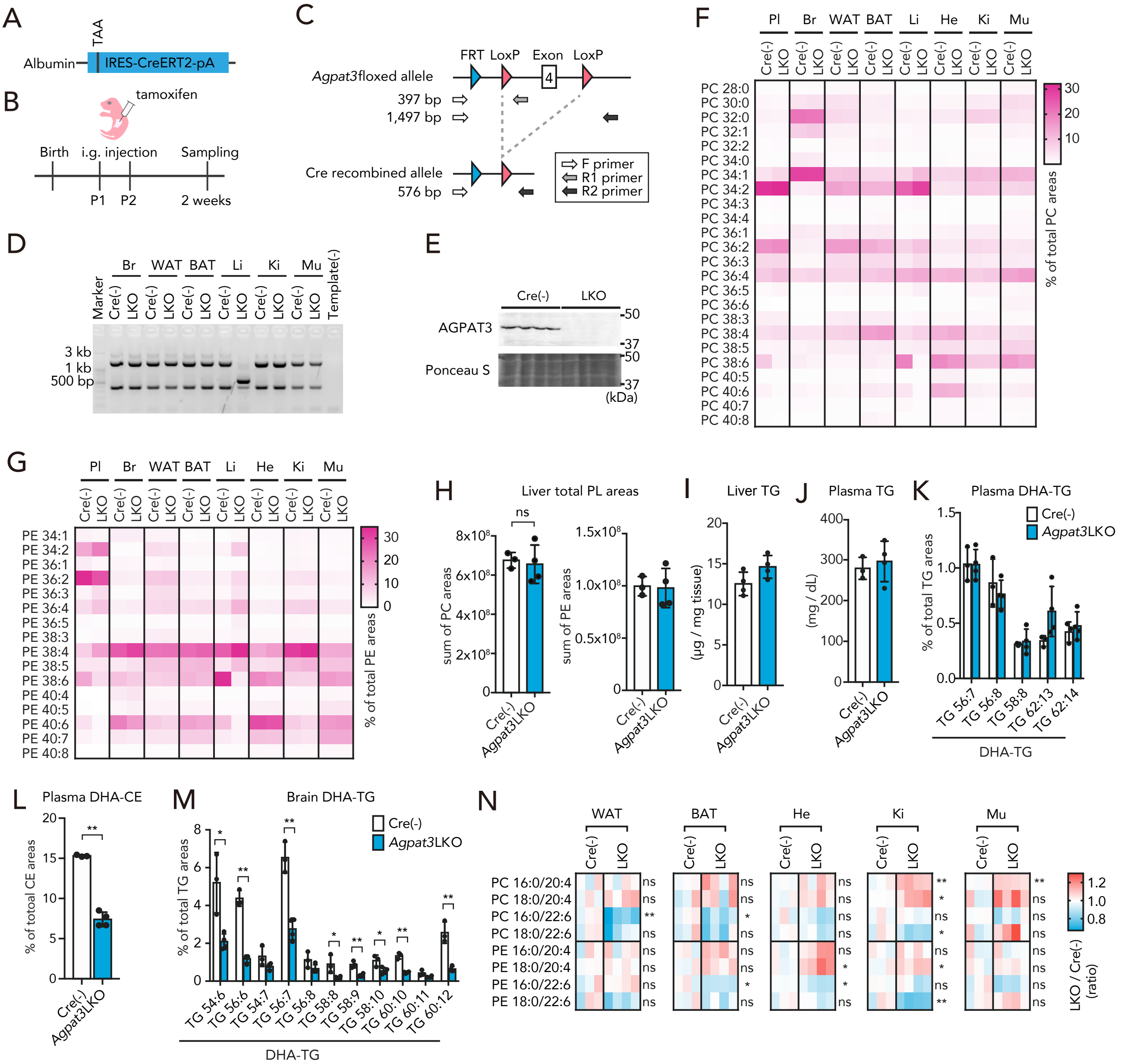

A
Albumin
TAA
IRES-CreERT2-pA
B
tamoxifen
Birth
i.g. injection
Sampling
P1
P2
2 weeks
C
FRT
LoxP
Exon
LoxP
Agpat3floxed allele
397 bp
1,497 bp
Cre recombined allele
576 bp
F primer
R1 primer
R2 primer
D
Marker
Br
WAT
BAT
Li
Ki
Mu
Template(-)
3 kb
1 kb
500 bp
E
Cre(-)
LKO
AGPAT3
Ponceau S
(kDa)
F
% of total PC areas
G
% of total PE areas
H
Liver total PL areas
sum of PC areas
sum of PE areas
ns
I
Liver TG
(μg / mg tissue)
J
Plasma TG
(mg / dL)
K
Plasma DHA-TG
% of total TG areas
Cre(-)
Agpat3LKO
DHA-TG
L
Plasma DHA-CE
% of total CE areas
M
Brain DHA-TG
% of total TG areas
DHA-TG
N
WAT
BAT
He
Ki
Mu
LKO / Cre(-) (ratio)

FIGURE S4

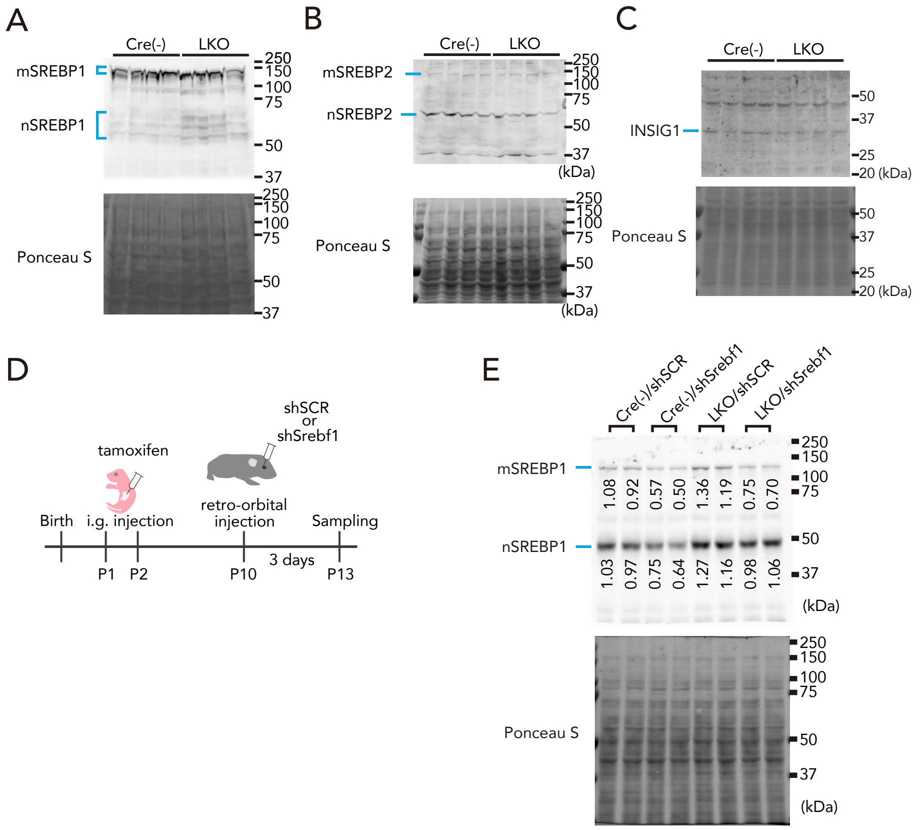
A
Cre(-)
LKO
mSREBP1
nSREBP1
250
150
100
75
50
37
Ponceau S
B
mSREBP2
nSREBP2
(kDa)
C
INSIG1
25
20 (kDa)
D
shSCR
or
shSrebf1
tamoxifen
Birth
i.g. injection
retro-orbital
injection
Sampling
3 days
P1
P2
P10
P13
E
Cre(-)/shSCR
Cre(-)/shSrebf1
LKO/shSCR
LKO/shSrebf1
1.08
0.92
0.57
0.50
1.36
1.19
0.75
0.70
1.03
0.97
0.75
0.64
1.27
1.16
0.98
1.06

FIGURE S5

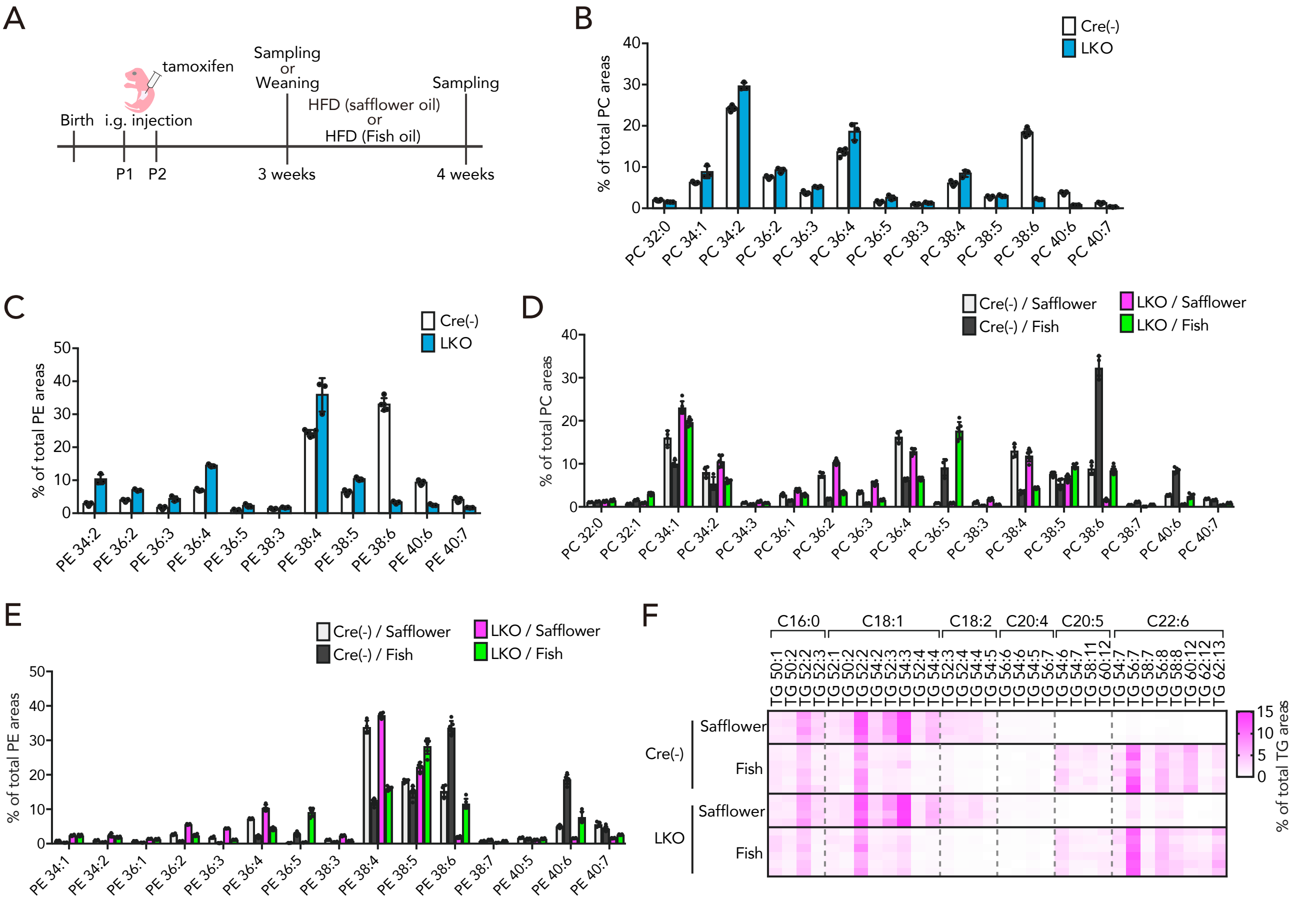
A
tamoxifen
Sampling
or
Weaning
Sampling
HFD (safflower oil)
or
HFD (Fish oil)
Birth
i.g. injection
P1
P2
3 weeks
4 weeks
B
Cre(-)
LKO
% of total PC areas
PC 32:0
PC 34:1
PC 34:2
PC 36:2
PC 36:3
PC 36:4
PC 36:5
PC 38:3
PC 38:4
PC 38:5
PC 38:6
PC 40:6
PC 40:7
C
Cre(-)
LKO
% of total PE areas
PE 34:2
PE 36:2
PE 36:3
PE 36:4
PE 36:5
PE 38:3
PE 38:4
PE 38:5
PE 38:6
PE 40:6
PE 40:7
D
Cre(-) / Safflower
Cre(-) / Fish
LKO / Safflower
LKO / Fish
% of total PC areas
PC 32:0
PC 32:1
PC 34:1
PC 34:2
PC 34:3
PC 36:1
PC 36:2
PC 36:3
PC 36:4
PC 36:5
PC 38:3
PC 38:4
PC 38:5
PC 38:6
PC 38:7
PC 40:6
PC 40:7
E
Cre(-) / Safflower
Cre(-) / Fish
LKO / Safflower
LKO / Fish
% of total PE areas
PE 34:1
PE 34:2
PE 36:1
PE 36:2
PE 36:3
PE 36:4
PE 36:5
PE 38:3
PE 38:4
PE 38:5
PE 38:6
PE 38:7
PE 40:5
PE 40:6
PE 40:7
F
C16:0
C18:1
C18:2
C20:4
C20:5
C22:6
TG 50:1
TG 50:2
TG 52:2
TG 52:3
TG 52:1
TG 50:2
TG 52:2
TG 54:2
TG 52:3
TG 54:3
TG 52:4
TG 54:4
TG 52:3
TG 52:4
TG 54:4
TG 54:5
TG 56:6
TG 54:6
TG 54:5
TG 56:7
TG 54:6
TG 54:7
TG 58:11
TG 60:12
TG 54:7
TG 56:7
TG 58:7
TG 56:8
TG 58:8
TG 60:12
TG 62:12
TG 62:13
Cre(-)
Safflower
Fish
LKO
Safflower
Fish
% of total TG areas

Supplemental Figure Legends

Figure S1. Phospholipid compositions of PUFA biosynthetic enzymes in the liver of *Agpat3* WT and KO mice, related to Figure 1.

(A-B) Percentages of each PC (A) and PE (B) species (10-week-old, n=3 for *Agpat3* WT and n=5 for *Agpat3* KO). Data are shown as means ± SD.

Figure S2. Phospholipid compositions and mRNA expression of DHA-related genes in mouse tissues, related to Figure 2.

(A-B) Relative mRNA expression of *Agpat3* in various tissues of 1-week-old (A) and 15-week-old (B) WT mice. (C-D) Heat maps show the percentages of each PC (C) and PE (D) species in plasma and tissues (n=3 for each group). Each column represents data for a mouse. (E-H) Relative mRNA expression of *Fads1*(E), *Fads2* (F), *Elovl2* (G), and *Elovl5* (H) in various tissues of 15-week-old WT and *Agpat3* KO mice. (A, B, and E-H) Data are shown as means ± SD. (E-H) Significance is based on two-way ANOVA followed by Bonferroni's post hoc test (*P < 0.05). Pl; plasma, Br; brain, WAT; white adipose tissue, BAT; brown adipose tissue, Li; liver, He; heart, Ki; kidney, SI; small intestine.

Figure S3. The effect of liver-specific *Agpat3* knockout on systemic lipid compositions, related to Figure 3.

(A) Scheme of Alb-CreERT2 allele. TAA; stop codon of endogenous *Albumin* gene, IRES; internal ribosome entry site, pA; polyadenylation site. (B) Scheme of *Agpat3* LKO mice generation. (C-D) Genotyping of *Agpat3* LKO mice. Primers and amplicon sizes (C), and example of PCR-based genotyping (D) are shown. (D-N) Samples were

prepared from 2-week-old mice. Cre(-) and LKO indicate control (Alb-CreERT2$^{-/-}$; *Agpat3*$^{fl/fl}$) and *Agpat3* LKO (Alb-CreERT2$^{+/-}$; *Agpat3*$^{fl/fl}$) mice, respectively. (E) Immunoblot analysis of AGPAT3 in the liver of Cre(-) and *Agpat3* LKO mice. Ponceau S staining was used as a loading control (n=4 for each group). (F and G) Heat map shows the average percentages of each PC (F) and PE (G) species (n=3 for Cre(-), n=4 for *Agpat3* LKO). Pl, plasma; Br, brain; WAT, white adipose tissue; BAT, brown adipose tissue; Li, liver; He, heart; Ki, kidney; Mu, muscle. (H) Total area values of PCs and PEs in the liver (n=3 for Cre(-), n=4 for *Agpat3* LKO). (I-J) TG levels in the liver (I, n=4 for each group) and plasma (J, n=3 for Cre(-), n=4 for *Agpat3* LKO). (K-M) Percentages of major DHA-TG species (K) and DHA-CE (L) in plasma, and major DHA-TG species in the brain (M) (n=3 for Cre(-), n=4 for *Agpat3* LKO). (N) Heat map shows the rational differences in the levels of each PL species in the brain (% of total area in Cre(-)/% of total area in LKO; n=3 for Cre(-) and n=4 for *Agpat3* LKO). Each column represents data for a mouse. (H-M) Data are shown as means ± SD. (H-N) Significance is based on unpaired *t*-test (*P < 0.05, **P < 0.01, ns; no significance).

Figure S4. SREBP1 induction in *Agpat3* LKO mice, related to Figure 4.

(A-C) Liver samples were prepared from 2-week-old (A and B) and 3-week-old (C) *Agpat3* LKO and Cre(-) mice. Immunoblot analysis of SREBP1 (A) and SREBP2 (B) and INSIG1 (C) using liver whole lysate. mSREBP and nSREBP indicate membrane-bound form and nuclear form of SREBP, respectively. Ponceau S staining was used as a loading control (n=4 for each group). (D) Scheme of in vivo gene knockdown experiment. shSCR, scrambled short hairpin RNA (shRNA); shSrebf1, *Srebf1* shRNA. (E) Liver samples were prepared from P13 *Agpat3* LKO and Cre(-) mice.

Immunoblot analysis of SREBP1 using liver whole lysate. Ponceau S staining was used as a loading control (n=2 for each group). Signal intensity of each band was quantified by Image J software and normalized by the average of Cre(-)/shSCR. Normalized values were indicated below the band.

Figure S5. The effect of fish oil- and safflower oil-containing HFD on lipid composition in the liver, related to Figure 5.

(A) Scheme of safflower oil- or fish oil-containing high fat diet (HFD)-fed experiment. (B-C) Percentages of each PC (B) and PE (C) species in the liver (3-week-old; n=5 for Cre(-) and n=3 for *Agpat3* LKO). (D-F) Percentages of each PC (D), PE (E) and TG (F) species in the liver of HFD-fed mice. Identified fatty acid in Q3 of LC-MS/MS analysis was indicated above an each TG species (n=4 for safflower-diet-fed Cre(-) and *Agpat3* LKO, and n=6 for fish-diet-fed Cre(-) and *Agpat3* LKO). Each column represents data for a mouse. (B-E) Data are shown as means ± SD.

Table S1. Fatty acid profile of safflower oil- and fish oil-containing HFD, related to Figure 5.

	Safflower-diet (D10031901)	Fish-diet (D18070301)
**Ingredient (gram%)**		
Protein	30.0	30.0
Carbohydrate	23.0	23.0
Fat	36.0	36.0
Beef fat	15.9	15.9
Safflower oil	20.0	(-)
Fish (Menhaden) oil	(-)	20.0
**Total**	100.0	100.0
**Fatty acid (gram)**		
C14:0	5.3	18.9
C14:1(n-9)	1.1	1.1
C15:0	0.8	1.7
C16:0	47.1	66.3
C16:1(n-9)	3.8	23.3
C16:2 (n-4)	0.0	3.2
C16:3(n-9)	0.0	3.0
C16:4(n-4)	0.0	3.1
C17:0	1.9	2.7
C18:0	29.1	30.9
C18:1(n-9)	217.0	80.2
C18:2(n-6)	33.6	9.0
C18:3(n-3)	0.2	3.2
C18:4(n-3)	0.0	6.2
C20:0	1.2	0.6
C20:1	0.9	3.3
C20:2	0.0	0.4
C20:3(n-6)	0.0	0.8
C20:4(n-6)	0.0	4.2
C20:5(n-3)	0.0	28.4
C21:5(n-3)	0.0	1.5
C20:0	0.0	0.2
C22:1	0.0	0.6
C22:4(n-6)	0.0	0.4
C22:5(n-3)	0.0	5.6
C22:6(n-3)	0.0	20.5
C24:0	0.0	1.2
C24:1	0.0	0.4
**Total (gram)**	342.0	320.9

Transparent Methods

Animals

All animal experiments were approved and performed in accordance with the guidelines of the Animal Research Committee of the National Center for Global Health and Medicine (19112, 19094). All experiments involving gene recombination were approved by and performed in accordance with the guidelines of the Biosafety Committee of the National Center for Global Health and Medicine (31-D-112).

Global and liver-specific *Agpat3* KO mice

Agpat3$^{tm1(EUCOMM)Wtsi}$ mice were obtained from the European Conditional Mouse Mutagenesis Program (EUCOMM) and used as *Agpat3* global KO mice. *Agpat3* floxed mice were generated by crossing *Agpat3*$^{tm1(EUCOMM)Wtsi}$ mice with C57BL/6-Tg(CAG-flpe)36Ito/ItoRbrc mice (RBRC01834, RIKEN, Japan) (Kanki et al., 2006) to delete *neo*^{r} casette. To generate tamoxifen-inducible *Agpat3* LKO mice, *Agpat3* floxed mice were crossed with Alb-Cre-ERT2 mice (Schuler et al., 2004). Alb-Cre-ERT2 mice were kindly provided by Pierre Chambon (IGBMC, Illkirch-Cedex, France) and Tsuneo Ikenoue (University of Tokyo, Japan). All mice used in this study were housed in an air-conditioned animal room at 23 ± 2°C and relative humidity of 40-60% under specific-pathogen-free (SPF) conditions, with a 12-h light/dark cycle (8:00-20:00/20:00-8:00). All mice were fed a standard rodent CE-2 diet (CLEA Japan, Tokyo, Japan) except for HFD-fed experiment, and had *ad libitum* access to water. In the HFD-fed experiment, safflower oil-based HFD (D10031901, Research Diets Inc., USA) or fish oil-based HFD (D18070301, Research Diets Inc., USA) was fed to mice.

Ingredients of both the HFD are summarized in Table S1. Two microliter of tamoxifen (25 mg/mL in ethanol, T5648, Sigma, USA) was intragastrically injected at postnatal day 1 and 2 to remove the exon 4 of *Agpat3*. The sequences of genotyping primers were as follows (5′-3′): *Agpat3* LKO primers (see also Figure S3C), F: GTAGAGGCTGGGTTCTGAGTTGC, R1: ACATCAAGTGTACCGCCTACTGC, R2: GGCCACCCCAGAAACTACTGAAG; primers for Cre recombinase (Ikenoue et al., 2016), F: GCATTACCGGTCGATGCAACGAGTGATGAG, R: GAGTGAACGAACCTGGTCGAAATCAGTGCG.

Annotation of lipids

Fatty acid species are denoted as CAA:B (C indicates “carbon,” AA and B indicate the number of carbons and double bonds, respectively). In the case of CAA:B(n-Y), Y indicates the position of the first double bond from the methyl-end (omega-end). Fatty acid composition of PC, PE, TG, and CE are denoted as X CC:D (X indicates the lipid class). CC and D indicate the sum of carbon numbers and double bonds in each lipid, respectively. In the case of fatty acid combinations, lipids are denoted as X EE:F/GG:H (EE and GG indicate the number of carbons and F and H indicate the number of double bonds in each fatty acid). The methods used in this study do not discriminate which *sn* position is present in each fatty acid residue.

Lipid extraction

Lipids were extracted from tissue homogenate (50 mg/mL in RLT buffer, which supplied in RNeasy Mini Kit, Qiagen, USA.) and plasma using the Bligh and Dyer method (Bligh and Dyer, 1959). Subsequently, lipids were dried with a centrifugal evaporator and

reconstituted in isopropyl alcohol. The obtained lipids were diluted and used for LC-MS/MS analysis.

LC-MS/MS (PC, lysoPC, and PE)

The fatty acid composition of PC, lysoPC, and PE was analyzed using the LC-MS/MS with multiple-reaction monitoring (MRM). LC-MS/MS analysis was performed using a Nexera UHPLC system and triple quadrupole mass spectrometer, LCMS-8050 (Shimadzu Corp., Japan). The extracted lipids were separated on an Acquity UPLC BEH C8 column (1.7 µm, 2.1 × 100 mm, Waters, USA) with a gradient of mobile phases A, B, and C (A: 5 mM NH_4HCO_3; B: acetonitrile; C: isopropyl alcohol) at 47 °C and a flow rate of 0.35 mL/min. The gradient for phospholipid analysis was as follows: time (% A/% B/% C): 0 min (50/45/5), 10 min (20/75/5), 20 min (20/50/30), 27.5 min (5/5/90), 28.5 min (5/5/90), 28.6 min (50/45/5). MRM transitions for each phospholipid were as follows (Q1, Q3): PC and lysoPC ($[M + H]^+$, 184.0), PE ($[M + H]^+$, neutral loss of 141). Fatty acid combinations of PC and PE were determined by additional MRM analyses with detection of fatty acid (FA) fragments at Q3 (Q1, Q3), PC ($[M + HCO_3]^-$, $[FA – H]^-$), and PE ($[M – H]^-$, $FA – H]^-$). The obtained column retention time of each PL was used for identification of the peaks of DHA-, EPA- and ARA-containing PLs.

LC-MS/MS (TG and CE)

The fatty acid composition of TG and CE was analyzed as described in previous studies (Koeberle et al., 2012) with minor modifications. TG and CE were analyzed by LC-MS/MS with MRM using LCMS-8060 (Shimadzu Corp.). The extracted lipids were separated on an Acquity UPLC BEH C8 column (1.7 µm, 2.1 × 100 mm, Waters) with a

gradient of mobile phases A, B, and C (A: 10 mM ammonium acetate/0.1% formic acid; B: ammonium acetate/0.1% formic acid/99% methanol; C: isopropyl alcohol) at 47 °C and a flow rate of 0.2 mL/min. The gradient for phospholipid analysis was as follows: time (% A/% B/% C): 0 min (30/20/50), 12 min (5/45/50), 15 min (10/10/80), 19 min (10/10/80), 19.1 min (30/20/50). For TG analysis, the combinations of following fatty acids were analyzed; C16:0, C16:1, C18:0-C18:3, C20:3-C20:5, and C22:4-C22:6. CE species esterified with C14:0, C14:1, C16:0, C16:1, C18:0-C18:3, C20:0-C20:5, and C22:0-C22:6. MRM transitions for each lipid were as follows: (Q1, Q3), TG ($[M + NH_4]^+$, neutral loss of FA and NH^3), CE ($[M + NH_4]^+$, 369.4).

GC-FID

The amount of each fatty acid species in the liver were quantified using GC-FID. Frozen liver tissues were pulverized using an automatic cryogenic pulverizer (Tokken, Japan). Methylation Reagent A from the Fatty acid Methylation Kit (16961-04, Nacalai Tesque, Japan) was directly added onto the pulverized tissues, and then C23:0 fatty acid stock solution was added to each sample as an internal standard. The fatty acids were then methylated using the Fatty Acid Methylation Kit following the manufacturer's instructions. The resulting fatty acid methyl esters (FAMEs) were purified using the Fatty Acid Methyl Ester Purification Kit (Nacalai Tesque, Japan). The FAME samples were evaporated and reconstituted in dichloromethane, and then analyzed using the GC-2010 Plus system (Shimadzu Corp.), equipped with an FID. The injector and detector temperatures were set to 240 and 250 °C, respectively. The flow rate for the carrier gas (Helium) was set at 45 cm/s linear velocity. FAME species were separated using the FAMEWAX column (12497, Restec, USA) by linear thermal gradient as follows

(temperature-raising rate (°C/min)): Start at 140 °C, 200 °C -11, 225 °C -3, 240 °C -20, and hold 240 °C for 5 min. FAME samples were identified and quantified using a mixture of the following fatty acid methyl ester standards: Supelco 37 component FAME Mix (Merck, Germany), C22:5(n-3)-FAME (Sigma), C22:5(n-6)-FAME (Nu-Chek Prep. Inc., USA), and C22:4(n-6)-FAME (Cayman, USA).

qPCR

Tissues were homogenized in RLT buffer (50 mg/mL) with Handy Micro Homogenizer (NS310E2, Microtec Co., Ltd., Japan). The homogenate was centrifuged at 12,000 × *g* and 4 °C for 10 min, and the supernatant was used for RNA extraction. Total RNA was extracted using the RNeasy Mini Kit (Qiagen, USA). Single-strand cDNA was synthesized using SuperScript III reverse transcriptase and random primers (Thermo Fisher Scientific, USA). qPCR was performed using the Fast SYBR Green Master Mix with the Step One Plus real-time PCR system (Applied Biosystems, USA). Relative mRNA expression levels were determined using the comparative cycle threshold method. Primer sequences (5′-3′) for qPCR were as follows: (Gene name: Forward, Reverse, Amplicon size (bp)). *Rplp0*: CTGAGATTCGGGATATGCTGTTG, AAAGCCTGGAAGAAGGAGGTCTT, (136); *Srebf1*: TGACCCGGCTATTTCCGTGA, CTGGGCTGAGCAATACAGTTC, (61); *Fads1*: GAAGAAGCACATGCCATACAACC, TCCGCTGAACCACAAAATAGAAA, (113); *Fads2*: GCCTGGTTCATCCTCTCGTACTT, GAAAGGTGGCCATAGTCATGTTG, (119); *Elovl2*: CCTGCTCTCGATATGGCTGG, AAGAAGTGTGATTGCGAGGTTAT, (100); *Elovl5*: ATGGAACATTTCGATGCGTCA, GTCCCAGCCATACAATGAGTAAG, (148); *Elovl6*: GAAAAGCAGTTCAACGAGAACG, AGATGCCGACCACCAAAGATA, (110); *Scd*: ACTGGTTCCCTCCTGCAAG,

GTGATCTCGGGCCCATTC, (199); *Fasn*: AGAGAAGAAAGCTGTGGCCCATG, AGCACCAGATCGTGTTCTCGTTC, (189); *Abca1*: GGGGTGGTGTTCTTCCTCATTAC, ACATCCTCATCCTCGTCATTCAA, (107); *G6pc*: CGACTCGCTATCTCCAAGTGA, GTTGAACCAGTCTCCGACCA (173); *Glut2*: TCAGAAGACAAGATCACCGGA, GCTGGTGTGACTGTAAGTGGG, (215); *Hmgcr*: AGCTTGCCCGAATTGTATGTG, TCTGTTGTGAACCATGTGACTTC, (104). The *Scd* primer was designed to detect all its major isoforms (*Scd1-4*). The *Srebf1* primer was designed to detect both *Srebf1a* and *Srebf1c*.

DNA microarray

Total RNA from the liver of adult mice was extracted using the RNeasy Mini Kit (Qiagen, USA). 100 ng of total RNA from *Agpat3* wild type (WT) and KO mice (n = 4, each) was examined using the SurePrint G3 Mouse GE 8x60K Microarray (Agilent Technologies, USA). Data were quantified using the Agilent Feature Extraction software (Agilent Technologies), and normalized using the GeneSpring software (Agilent Technologies). The highly expressed genes in the *Agpat3* KO mice, with fold change > 1.5 and $P < 0.2$, were analyzed for common functions of altered genes using gene ontology (GO) terms by employing Database for Annotation, Visualization, and Integrated Discovery (DAVID) (https://david.ncifcrf.gov/).

Measurement of TGs

The amount of TGs in the liver and plasma was determined using the LabAssay Triglyceride Kit (Wako, Japan). Plasma was directly used for the measurement. For the measurement of TGs in the liver, tissue homogenate was used. Liver was homogenized

in RIPA buffer (1 M TrisHCl (pH 7.4), 150 mM NaCl, 0.5% sodium deoxycholate, 0.1% sodium dodecyl sulfate, 1% NP-40) with a cOmplete protease inhibitor cocktail (Sigma) at a concentration of 100 mg/mL. The homogenate was diluted 5-fold with 5% NP-40. The diluted samples were boiled at 100 °C for 5 min and cooled at room temperature; this was repeated twice. Thereafter, the samples were centrifuged at 3,000 × *g* for 2 min at 25 °C, and the supernatant was used for the TG assay.

Preparation of nuclear extract from liver samples

The nuclear extract from liver samples was prepared as described previously (Sheng et al., 1995) with a small modification. Freshly isolated liver (100 mg) was homogenized in 1 mL of buffer A (10 mM HEPES (pH 7.6), 25 mM KCl, 1 mM EDTA (pH 8.0), 2 M sucrose, 10% glycerol, 0.15 mM spermine, 2 mM spermidine) with a cOmplete protease inhibitor cocktail using Potter-Elvehjem Tissue Grinder (Wheaton, USA). Tissue homogenate was layered over 300 µL of buffer A and centrifuged at 85,000 × *g* for 1 hour at 4 °C using a swinging bucket TLS 55 Rotor (Beckman, USA). The resulting nuclear pellet was resuspended in 200 µL of RIPA buffer and incubated on ice for 30 min. During 30 min incubation, the sample was mixed every 5 min. Thereafter, the sample was centrifuged at 100,000 × *g* for 30 min at 4 °C using the S55A2 rotor (Hitachi, Japan). The supernatant was used as the liver nuclear extract. The protein concentration was determined using the Pierce BCA Protein Assay kit (Thermo Fisher Scientific) and used for immunoblot analyses.

Preparation of whole liver lysate

The liver was homogenized in RIPA buffer (1 M TrisHCl (pH 7.4), 150 mM NaCl, 0.5%

sodium deoxycholate, 0.1% sodium dodecyl sulfate, 1% NP-40) with a cOmplete protease inhibitor cocktail using Handy Micro Homogenizer at a concentration of 100 mg/mL. The samples were centrifuged at 12,000 × *g* for 10 min at 4 °C. The resulting supernatant was used as the whole liver lysate. The protein concentration was determined using the Pierce BCA Protein Assay kit and used for immunoblot analyses.

Immunoblot

Protein samples were resolved on 10% (for AGPAT3, PPARα, and INSIG1) or 8% (for SREBP1 and SREBP2) SDS-polyacrylamide gels and transferred onto nitrocellulose membranes (GE Healthcare, USA) using Trans-Blot Turbo (BioRad, USA). The membranes were stained with Ponceau S (Sigma) to visualize the total protein levels. After destaining the membranes, they were blocked with the blocking buffer (5% skimmed milk in Tris-buffered saline, with 0.1% polyoxyethylene(20) sorbitan monolaurate (Wako)) at room temperature for 1 h. The anti-AGPAT3 (Koeberle et al., 2012), anti-SREBP1 (sc-13551, Santa Cruz, USA), anti-SREBP2 (ab30682, Abcam, UK), anti-PPARα (sc-398394, Santa Cruz), and anti-INSIG1 (ab70784, Abcam, UK) antibodies were used in this study. The membrane was incubated with the primary antibody at 4 °C for 16 h, washed three times with the wash buffer (Tris-buffered saline with 0.1% polyoxyethylene(20) sorbitan monolaurate) for 10 min each, and then incubated with anti-mouse (for SREBP1 and PPARα) or anti-rabbit (for AGPAT3 and SREBP2) IgG antibody conjugated to horseradish peroxidase (GE Healthcare) at room temperature for 1 h. The membranes were washed with the wash buffer three times for 10 min each, and developed with the ECL reagent (GE Healthcare). Immunoreactive proteins were visualized using ImageQuant LAS 500 (GE Healthcare, USA). The

protein bands corresponding to SREBP1 were manually selected and quantified with the Wand Tool in the ImageJ software (version: 2.0.0-rc-69/1.52p). The signal intensities from different membranes were normalized relative to the control samples.

In vivo shRNA injection

Adenoviruses carrying *Srebf1* shRNA and scramble (control) shRNA under U6 promoter (> 10^{12} viral particles/mL in 2.5% glycerol/20 mM TrisHCl pH 8.0/25 mM NaCl) were purchased from VectorBuilder (VectorBuilder Inc., USA.). The target sequence of *Srebf1* and scramble shRNAs were CATCTGTTGTAAGGTGTATTT and CCTAAGGTTAAGTCGCCCTCG, respectively. The *Srebf1* shRNA were targeted to the 3′-untranslated region of the *Srebf1* gene. Adenovirus was diluted 10-fold with phosphate-buffered saline (pH 7.4), and then retro-orbitally injected (50 μL) into mice at postnatal day 10. Liver samples were collected at postnatal day 13 (72 h after injection).

Statistical analyses

Unpaired *t*-tests were used when two groups were compared. When two factors were present, two-way ANOVA test was performed. Bonferroni's post hoc test was used when ANOVA showed a significant difference. All statistical analyses were performed using the GraphPad Prism 7 (version 7.0d) software.

Supplemental References

Bligh, E.G., and Dyer, W.J. (1959). A rapid method of total lipid extraction and purification. Can J Biochem Physiol *37*, 911-917.

Ikenoue, T., Terakado, Y., Nakagawa, H., Hikiba, Y., Fujii, T., Matsubara, D., Noguchi, R., Zhu, C., Yamamoto, K., Kudo, Y., et al. (2016). A novel mouse model of intrahepatic cholangiocarcinoma induced by liver-specific Kras activation and Pten deletion. Sci Rep *6*, 23899.

Kanki, H., Suzuki, H., and Itohara, S. (2006). High-efficiency CAG-FLPe deleter mice in C57BL/6J background. Exp Anim *55*, 137-141.

Koeberle, A., Shindou, H., Harayama, T., Yuki, K., and Shimizu, T. (2012). Polyunsaturated fatty acids are incorporated into maturating male mouse germ cells by lysophosphatidic acid acyltransferase 3. FASEB J *26*, 169-180.

Schuler, M., Dierich, A., Chambon, P., and Metzger, D. (2004). Efficient temporally controlled targeted somatic mutagenesis in hepatocytes of the mouse. Genesis *39*, 167-172.

Sheng, Z., Otani, H., Brown, M.S., and Goldstein, J.L. (1995). Independent regulation of sterol regulatory element-binding proteins 1 and 2 in hamster liver. Proc Natl Acad Sci U S A *92*, 935-938.
